# Back Interface and Absorber Bulk Deep‐Level Trap Optimization Enables Highly Efficient Flexible Antimony Triselenide Solar Cell

**DOI:** 10.1002/advs.202310193

**Published:** 2024-03-20

**Authors:** Jia Yang, Mingdong Chen, Guojie Chen, Yanqing Hou, Zhenghua Su, Shuo Chen, Jun Zhao, Guangxing Liang

**Affiliations:** ^1^ State Key Laboratory of Complex Non‐ferrous Metal Resources Clean Utilization Kunming University of Science and Technology Kunming 650093 China; ^2^ Shenzhen Key Laboratory of Advanced Thin Films and Applications Key Laboratory of Optoelectronic Devices and Systems of Ministry of Education and Guangdong Province College of Physics and Optoelectronic Engineering Shenzhen University Shenzhen Guangdong 518060 China

**Keywords:** Back interface, Defect, Efficiency, Flexible solar cell, Sb_2_Se_3_

## Abstract

The unique 1D crystal structure of Antimony Triselenide (Sb_2_Se_3_) offers notable potential for use in flexible, lightweight devices due to its excellent bending characteristics. However, fabricating high‐efficiency flexible Sb_2_Se_3_ solar cells is challenging, primarily due to the suboptimal contact interface between the embedded Sb_2_Se_3_ layer and the molybdenum back‐contact, compounded by complex intrinsic defects. This study introduces a novel Molybdenum Trioxide (MoO_3_) interlayer to address the back contact interface issues in flexible Sb_2_Se_3_ devices. Further investigations indicate that incorporating a MoO_3_ interlayer not only enhances the crystalline quality but also promotes a favorable [hk1] growth orientation in the Sb_2_Se_3_ absorber layer. It also reduces the barrier height at the back contact interface and effectively passivates harmful defects. As a result, the flexible Sb_2_Se_3_ solar cell, featuring a Mo‐foil/Mo/MoO_3_/Sb_2_Se_3_/CdS/ITO/Ag substrate structure, demonstrates exceptional flexibility and durability, enduring large bending radii and multiple bending cycles while achieving an impressive efficiency of 8.23%. This research offers a straightforward approach to enhancing the performance of flexible Sb_2_Se_3_ devices, thereby expanding their application scope in the field of photovoltaics.

## Graphical abstract

A MoO_3_ interlayer is introduced to optimize back contact and passivate detrimental defects. A high efficiency of 8.23% can be achieved in flexible Sb_2_Se_3_ thin‐film solar cell, accompanied with superior flexibility.

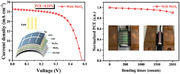

## 1 Introduction

Recent decades have witnessed remarkable progress in photovoltaic (PV) technology, addressing humanity's energy demands while mitigating environmental impact.^[^ ^1^ ^]^ Among various solar cell types, thin‐film solar cells are particularly notable, offering minimal material usage, light weight, flexibility, and scalability.^[^ ^2^, ^3^, ^4^ ^]^ These features render them ideal for diverse applications, including portable devices, Internet of Things (IoT) integration, and building‐integrated photovoltaic (BIPV) systems.^[^ ^5^, ^6^ ^]^ With technological and industrial advancements, there's an increasing need for flexible micro‐electronic energy solutions. This urgency underscores the importance of ongoing research into sustainable, efficient, and stable thin‐film solar cells. Identifying and utilizing eco‐friendly, cost‐effective, inherently stable materials with superior photoelectric properties for high‐efficiency flexible thin‐film solar cells is therefore a priority.

Antimony Triselenide (Sb_2_Se_3_) has emerged as a viable thin‐film photovoltaic (TFPV) material, owing to its earth‐abundance, low toxicity, and exceptional stability.^[^ ^7^, ^8^, ^9^ ^]^ It exhibits superior optoelectronic characteristics, including an ideal bandgap range of 1.1–1.3 eV, a high absorption coefficient (≈10^5^ cm^−1^ at short wavelengths), satisfactory carrier mobility (≈10 cm^2^ V^−1^ s^−1^), and a long carrier lifetime (≈60 ns).^[^ ^10^, ^11^ ^]^ Its unique 1D crystal structure, formed by (Sb_4_Se_6_)_n_ ribbons connected through robust Sb─Se covalent bonds and weaker van der Waals forces, confers notable resistance to deformation and electrically favorable grain boundaries. This makes it particularly suitable for flexible device applications.^[^ ^12^, ^13^, ^14^ ^]^ Theoretical models suggest Sb_2_Se_3_’s fracture strain could reach 28%, comparable to some ductile organic polymers.^[^ ^12^, ^15^ ^]^ While rigid Sb_2_Se_3_ devices have achieved efficiencies over 10.5%,^[^ ^16^ ^]^ research on their flexible counterparts is less advanced, with the highest efficiency recorded at 8.43%.^[^ ^17^ ^]^ This indicates a significant performance gap and highlights the need for more research to enhance the efficiency of flexible Sb_2_Se_3_ devices.

The progression of flexible Sb_2_Se_3_ solar devices is closely linked to advancements in flexible substrates and device architectures.^[^ ^18^, ^19^ ^]^ Presently, planar flexible Sb_2_Se_3_ devices are predominantly classified into superstrate and substrate configurations.^[^ ^8^, ^20^ ^]^ In superstrate configurations, such as those using polyimide (PI) substrates, light penetration through the substrate is essential for cell activation. However, the PI substrate's limited short‐wavelength transmittance can result in significant current loss, thereby reducing the device's power conversion efficiency (PCE) in comparison to glass‐based substrates.^[^ ^15^ ^]^ In contrast, substrate‐configured flexible solar cells are less dependent on the optical properties of the substrate, provided it remains stable throughout the fabrication process.^[^ ^21^ ^]^ Nonetheless, research on substrate‐configured Sb_2_Se_3_ thin‐film solar cells, particularly on flexible substrates, is scarce. In 2018, Wang et al. reported the first instance of such cells on molybdenum (Mo) foil, achieving 5.9% efficiency.^[^ ^18^ ^]^ A notable deficiency in open‐circuit voltage (*V*_OC_) and fill factor (FF) contributes significantly to the efficiency shortfall in flexible Sb_2_Se_3_ devices. Research indicates that the quality of the back‐contact interface is critical for the growth of the Sb_2_Se_3_ absorber layer and the back‐contact barrier, both imperative for effective carrier transport and extraction.^[^ ^22^, ^23^, ^24^, ^25^ ^]^ To enhance this interface, several groups have explored using PbS colloidal quantum dots, NiO_x_, or CuSCN films as hole transport layers in rigid configurations, thereby improving carrier transport and minimizing contact barriers.^[^ ^26^, ^27^, ^28^ ^]^ Additionally, Mai's group has employed MoSe_2_ or PbSe layers to refine the Mo/Sb_2_Se_3_ interface, boosting carrier collection efficiency.^[^ ^17^, ^25^ ^]^ Liang's group, achieving a PCE of 9.24% in rigid substrate‐structured Sb_2_Se_3_ solar cells, employed effective interface engineering.^[^ ^24^ ^]^ They also introduced a NaF intermediate layer for back contact optimization and defect mitigation, successfully fabricating flexible Sb_2_Se_3_ thin‐film solar cells with efficiencies exceeding 8%.^[^ ^29^ ^]^ Therefore, interface engineering emerges as a vital strategy for enhancing Sb_2_Se_3_ device performance, necessitating further improvements, especially in the back contact interface of flexible devices.

Recently, MoO_3_ thin film, as a wide‐bandgap material with high work function, has been widely used as an interfacial layer in CIGS, CZTSSe, and CdTe solar cells to improve the transmission channel for holes and lessen the carrier recombination at back contact region.^[^ ^30^, ^31^ ^]^ In this study, we introduce a methodology to enhance the efficiency of flexible Sb_2_Se_3_ solar cells via back‐contact interface engineering in a substrate configuration. A MoO_3_ interlayer is deposited onto flexible Mo‐foil by magnetron sputtering before preparing the Sb precursor layer. Our findings reveal that the MoO_3_ interlayer not only enhances the crystalline quality and fosters a beneficial [hk1] growth orientation in Sb_2_Se_3_ but also effectively reduces the back contact barrier height. This facilitates efficient transport and collection of photogenerated carriers at the buried interface while simultaneously reducing deep defect density and suppressing carrier recombination. Consequently, the flexible Sb_2_Se_3_ solar cell achieves an impressive PCE of 8.23%, marked by a substantial increase in *V*_OC_ (0.479 V) and FF (64.93%). Moreover, the device exhibits remarkable stability and flexibility, withstanding large bending radii and multiple bending cycles. Our study thus presents a robust approach to fabricating highly efficient and flexible Sb_2_Se_3_ photovoltaics.

## 2 Results and Discussion

**Figure 1:**
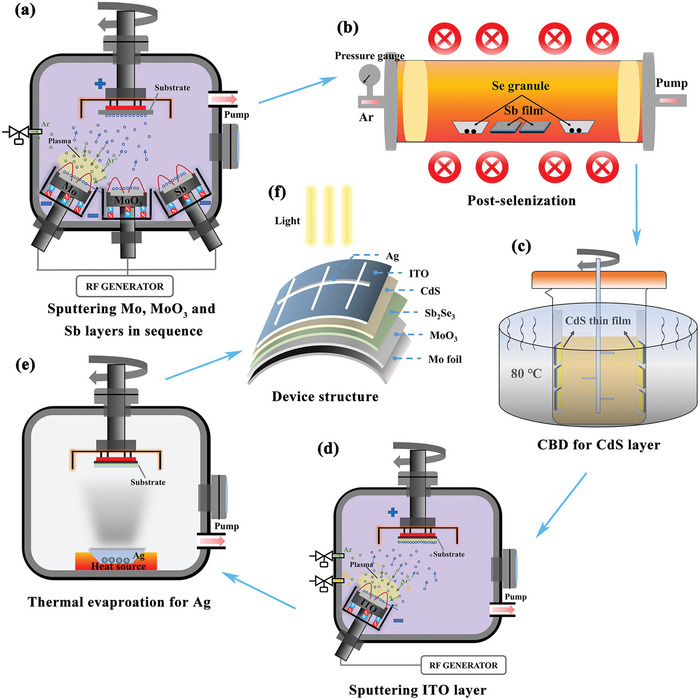
Schematic diagram of the the substrate structured flexible Sb_2_Se_3_ solar cell fabrication. a) Mo, MoO_3_, and Sb layer deposited by magnetron sputtering in sequence. b) Post‐selenization heat treatment of Sb precursor film. c) CBD for CdS buff layer. d) ITO layer deposited by magnetron sputtering. e) Ag electrode prepared via thermal evaporation process. f) The structure of final flexible Sb_2_Se_3_ solar cells.

To enhance the back contact interface between Sb_2_Se_3_ and flexible Mo‐foil carbon, a thin MoO_3_ interlayer was meticulously deposited onto the pre‐fabricated Mo‐foil/Mo thin film using magnetron sputtering, as depicted in the schematic in **Figure** **1**a. Detailed sputtering parameters for MoO_3_ deposition are provided in the Experimental Section. This flexible device structure was completed by subsequent layering of Sb_2_Se_3_ absorber, CdS buffer, ITO window layers, and Ag grids, corresponding to a Mo/MoO_3_/Sb_2_Se_3_/CdS/ITO/Ag substrate configuration shown in Figure 1.

**Figure 2:**
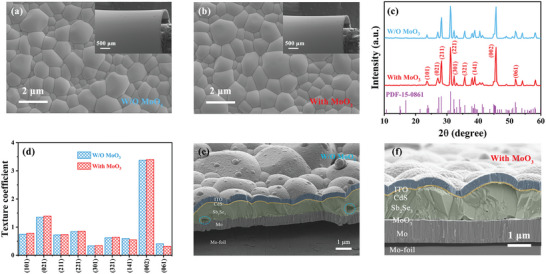
Top‐view SEM images of the Sb_2_Se_3_ thin films deposited on flexible Mo‐foil substrates: a) without MoO_3_ and b) with MoO_3_ interlayer, labeled W/O MoO_3_ and With MoO_3_, respectively. The Sb_2_Se_3_ thin films were bending at a 3 mm radius. c) XRD patterns of W/O MoO_3_ and With MoO_3_ Sb_2_Se_3_ thin films. d) Texture coefficients of the diffraction peaks of the W/O MoO_3_ and With MoO_3_ thin films. Cross‐sectional SEM image of the e) W/O MoO_3_ and f) With MoO_3_ Sb_2_Se_3_ solar cells.

Our initial investigation involved SEM analysis to compare the surface morphology of Sb_2_Se_3_ thin films on flexible Mo‐foil substrates with and without the MoO_3_ layer. For clarity, samples without and with the MoO_3_ layer are referred to as W/O MoO_3_ and With MoO_3_, respectively. As seen in **Figure** **2**a,b, both types of films, even when bent to a 3 mm radius, displayed similar morphologies without any microscale cracks, highlighting Sb_2_Se_3_’s remarkable flexibility. Moreover, frequency histograms against grain size distribution (Figure S1, Supporting Information) revealed that the MoO_3_ interlayer led to increased grain size in the Sb_2_Se_3_ thin films, suggesting enhanced compactness and uniformity. The crystallization characteristics of the Sb_2_Se_3_ films were further elucidated through XRD analysis. The patterns, as shown in Figure 2c, were in excellent agreement with the orthorhombic phase of Sb_2_Se_3_, free from impurities or by‐products. Notably, the films exhibited preferred growth orientations, as confirmed by distinct diffraction peaks. The texture coefficients (TCs) for these orientations were calculated (details in Note S1, Supporting Information), with results in Figure 2d indicating that the presence of the MoO_3_ layer notably enhanced the TCs of the [hk1] planes, thereby improving film crystallinity and carrier transport efficiency. Raman spectroscopy of both W/O MoO_3_ and With MoO_3_ samples (Figure S2, Supporting Information) further supported these findings, revealing characteristic peaks of Sb_2_Se_3_’s bending vibrations. Cross‐sectional SEM images (Figure 2e,f) contrasted the back contact quality between the two types of devices. The With MoO_3_ device showed a densely packed layer of large, well‐oriented crystal grains, in stark contrast to the W/O MoO_3_ device, which suffered from poor back contact quality, as indicated by visible holes. In conclusion, these results demonstrate that the introduction of a MoO_3_ interlayer via sputtering significantly enhances carrier transport and reduces recombination in Sb_2_Se_3_ films, promoting the preferred [hk1] orientation and improving back contact quality.

**Figure 3:**
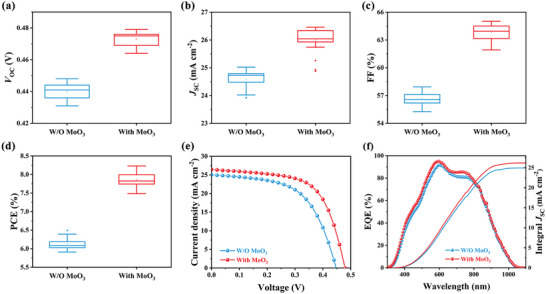
Statistical distribution of the key performance parameters of the flexible Sb_2_Se_3_ thin‐film solar cells, including a) *V*_OC_, b) *J*_SC_, c) FF, and d) PCE. e) *J*–*V* curves of the W/O MoO_3_ and With MoO_3_ devices. f) EQE and integrated *J*_SC_ of the flexible devices.

In our study, the MoO_3_ interlayer's optimal thickness was determined to be ≈5 nm, as this yielded the best performance in our devices (**Figures** **3** and S3, Supporting Information). We then proceeded to investigate the impact of the MoO_3_ interlayer on flexible Sb_2_Se_3_ solar cell performance. This involved preparing two sets of flexible Sb_2_Se_3_ solar cells, with 25 devices in each set, for comprehensive testing. The statistical distributions of crucial performance metrics such as open‐circuit voltage (*V*_OC_), short‐circuit current density (*J*_SC_), fill factor (FF), and power conversion efficiency (PCE) are depicted in Figure 3a–d. Notably, devices with the MoO_3_ interlayer exhibited substantial improvements in *V*_OC_ and FF when compared to those without it. The average *V*_OC_ and FF for devices with the MoO_3_ interlayer were elevated to 0.475 V and 64%, respectively. This enhancement is primarily attributed to the improved quality of the absorber layer and interfaces, including the back contact and heterojunction interfaces (Figure 3a,c). The current density–voltage (*J*–*V*) characteristics of both sets of devices are presented in Figure 3e. The device without the MoO_3_ interlayer reached a maximum PCE of 6.49%, with a *V*_OC_ of 0.448 V, a *J*_SC_ of 25.02 mA cm^−2^, and an FF of 57.93%. In contrast, the device with the MoO_3_ interlayer achieved an impressive PCE of 8.23%, along with a *V*_OC_ of 0.479 V, a *J*_SC_ of 26.46 mA cm^−2^, and an FF of 64.93%. This enhanced photovoltaic performance can be ascribed to the optimized back contact and the effective passivation of detrimental defects, which significantly reduce non‐radiative recombination losses in carriers. **Table** **1** provides a comparative analysis with other flexible Sb_2_Se_3_ devices reported in the literature.^[^ ^14^, ^15^, ^17^, ^18^, ^29^, ^32^ ^]^ The devices we developed with the MoO_3_ interlayer demonstrate competitive performance, especially in terms of *V*_OC_ and FF. Moreover, external quantum efficiency (EQE) and integrated *J*_SC_ for both types of devices are shown in Figure 3f. The devices with the MoO_3_ interlayer showed slight improvements in EQE response across the 350–1000 nm wavelength range, likely due to the MoO_3_ interlayer's role in defect center passivation within the absorber layer and enhanced carrier collection efficiency. The integrated *J*_SC_ values for devices without and with the MoO_3_ layer are 24.94 and 26.19 mA cm^−2^, respectively, aligning with the *J*_SC_ values derived from the *J*–*V* curves. Additionally, the bandgap (*E*_g_) for both types of devices was calculated from the EQE curves (Figure S4, Supporting Information), found to be 1.227 eV for devices without MoO_3_ and 1.225 eV for those with MoO_3_.

**Table 1:** A comparison of photovoltaic parameters with state‐of‐the‐art flexible Sb_2_Se_3_ devices.

Substrate	Device configuration	PCE [%]	*V*_OC_ [V]	*J*_SC_ [mA cm^−2^]	FF [%]	References
Mo‐foil	Mo/Sb_2_Se_3_/In_2_S_3_/ZnO/ITO	5.35	0.370	28.22	51.90	[18]
PI	ITO/CdS/Sb_2_Se_3_/Au	6.13	0.415	25.50	58.00	[15]
Photoresist	ITO/CdS/Sb_2_Se_3_/Au	7.15	0.426	29.30	57.30	[30]
PI	Mo/PbSe/Sb_2_Se_3_/CdS/ZnO/AZO/Ag	8.43	0.452	29.00	64.30	[17]
Mica	Mo/Sb_2_Se_3_/CdS/ITO/Ag	8.42	0.470	31.30	57.30	[14]
Mo‐foil	Mo/NaF/Sb_2_Se_3_/CdS/ITO/Ag	8.03	0.492	26.21	62.30	[29]
Mo‐foil	Mo/MoO_3_/Sb_2_Se_3_/CdS/ITO/Ag	8.23	0.479	26.46	64.93	This work

**Figure 4:**
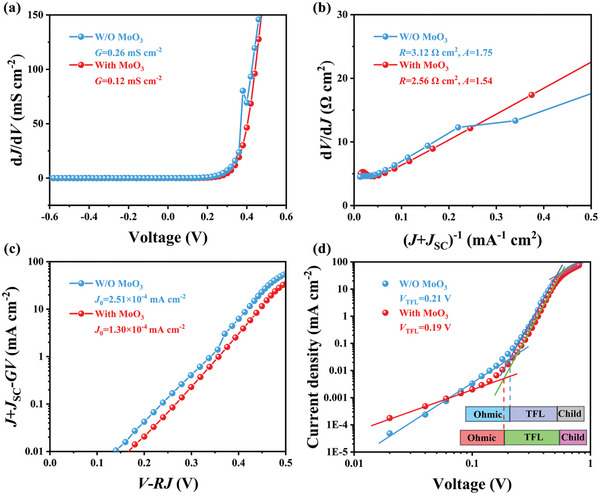
Electrical behaviors of the W/O MoO_3_ and With MoO_3_ devices: a) shunt conductance *G* characterizations, b) series resistance *R* and ideality factor *A* characterizations, c) reverse saturation current density *J*_0_ characterizations. d) The logarithmic *J*–*V* curves of the W/O MoO_3_ and With MoO_3_ devices, respectively, showing Ohmic, TFL, and Child region.

To elucidate the carrier recombination dynamics in flexible Sb_2_Se_3_ solar cells, we conducted dark current–voltage (*J*–*V*) measurements on representative devices both with and without the MoO_3_ interlayer. Detailed methodologies for these calculations are provided in Note S2 (Supporting Information). We first estimated the shunt conductance (*G*) values from the d*J*/d*V* versus *V* plots under reverse bias conditions, as shown in **Figure** **4**a. The calculated *G* values were 0.26 mS cm^−2^ for devices without MoO_3_ and 0.12 mS cm^−2^ for those with MoO_3_. Further analysis involved examining the d*V*/d*J* versus (*J*+*J*_SC_)^−1^ plots (Figure 4b), which allowed us to determine the series resistance (*R*) from the *y*‐axis intercept and the diode ideality factor (*A*) from the slope, representing *AkT/q*. The linear fitting of *R* values yielded 3.12 Ω cm^2^ for devices without MoO_3_ and 2.56 Ω cm^2^ for those with MoO_3_. The calculated *A* values were 1.54 and 1.75, respectively, with the lower *A* value in the MoO_3_‐enhanced devices indicating a reduction in space‐charge region (SCR) recombination losses. The reverse saturation current (*J*_0_) was derived from the ln(*J*+*J*_SC_−*GV*) versus *V−RJ* plot (Figure 4c), revealing *J*_0_ values of 2.51 × 10^−4^ mA cm^−2^ for devices without MoO_3_ and 1.30 × 10^−4^ mA cm^−2^ for those with MoO_3_. The reduced *J*_0_ in the latter suggests a successful diminution in interfacial recombination losses, attributable to the presence of the MoO_3_ layer. Additionally, space charge limited current (SCLC) analysis provided deeper insights into the defect states of these devices.^[^ ^33^ ^]^ The logarithmic *J*–*V* characteristic curves (Figure 4d) identified three distinct regions: the ohmic region at low voltages (exponent n = 1), the trap‐free Child region at high voltages (n > 2), and the trap‐filled limit (TFL) region at intermediate voltages (n > 3). The onset voltages in the TFL region (*V*_TFL_) were found to be 0.21 V for devices without MoO_3_ and 0.19 V for those with MoO_3_. The trap state density (*N*_trap_) can be calculated via the following equation^[^ ^34^, ^35^ ^]^

(1) $$\;N_{\mathrm{trap}}=\frac{2\varepsilon_{0}\varepsilon_{r}V_{\mathrm{TFL}}}{L^{2}q}\;$$

In our equation, *q* represents the elementary charge, *L* is the thickness of the Sb_2_Se_3_ layer, *ε*_0_ denotes the vacuum permittivity, and *ε*_r_ stands for the relative permittivity. The calculated trap state densities (*N*_trap_) showed a reduction from 1.55 × 10^14^ cm^−3^ in devices without MoO_3_ to 1.40 × 10^14^ cm^−3^ in devices with MoO_3_. This decrease suggests that the Sb_2_Se_3_ absorber layer with the MoO_3_ interlayer has fewer trap sites and defect recombination centers, likely due to the Mo/Sb_2_Se_3_ interface enhancement from the MoO_3_ treatment, thereby improving the quality of the absorber layer.

Furthermore, we explored how the current density–voltage characteristics (*J*–*V*–*T*) vary with temperature, ranging from 200 to 300 K under dark conditions (Figure S5, Supporting Information). This analysis aimed to deepen our understanding of how the MoO_3_ interlayer contributes to the improvement of fill factor (FF) and power conversion efficiency (PCE). Typically, low FF in chalcogenide solar cells, especially those based on molybdenum, is attributed to high dark series resistance (*R*_S,D_).^[^ ^29^ ^]^ The *R*_S,D_ can be calculated using the following relationship, as detailed in reference:^[^ ^36^ ^]^

(2) $$dV/dJ\;=R_{S,D}\;+AkT/q\left(J-G_{D}V\right)$$

**Figure 5:**
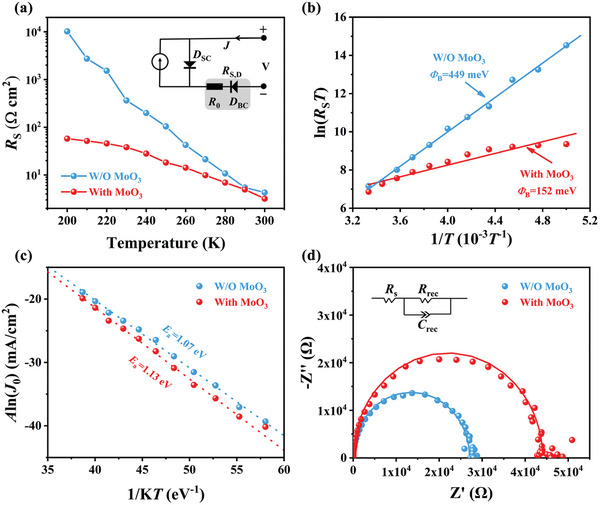
a) Temperature‐dependent *R*_S,D_ of the representative W/O MoO_3_ and With MoO_3_ flexible devices, and a simple circuit model of the thin‐film solar cell (insert). b) Blocking contact barrier height determination of the devices. c) *Aln*(*J*_0_) versus *1*/*kT* plots, wherein, *E*_a_ values can be estimated from the slopes. d) Electrochemical recombination analysis in the devices. Nyquist plots of W/O MoO_3_ and With MoO_3_ devices. The inset shows the equivalent circuit diagram.

In this section, “*A*” denotes the ideality factor, “*k*” the Boltzmann constant, and “*G*_D_” the dark shunt conductance. The dark series resistance (*R*_S,D_) is ascertained by analyzing the *y*‐intercept in **Figure** **5**a. A notable trend observed is the increase in *R*_S,D_ for the device without MoO_3_ as temperature decreases, in contrast to a smaller magnitude increase for the device with MoO_3_. To model the flexible device, a Schottky diode is used to represent the back contact junction. The circuit model incorporates a primary diode (*D*_SC_) and a combined *R*_S,D_, which includes both the background series resistance (*R*_0_) and the back contact diode (*D*_BC_).^[^ ^37^ ^]^ This model accounts for changes in *R*_S,D_ with varying temperatures in the device.

(3) $$\;R_{S,D}=R_{0}\;+\frac{k}{qA^{*}T}\times \exp\left(\frac{\Phi_{B}}{kT}\right)$$

In this study, A* signifies the effective Richardson constant, while *Φ*_B_ represents the blocking contact barrier height. As illustrated in Figure 5b, there is a substantial reduction in *Φ*_B_ for the device with MoO_3_, decreasing from 449 to 152 meV. This indicates an improvement in ohmic contact, which enhances carrier transport and collection at the back contact interface. This finding correlates with the observed decrease in series resistance and increased fill factor in devices with MoO_3_. Dark *J*–*V*‐*T* measurements further illuminate the recombination mechanisms in these flexible Sb_2_Se_3_ devices, especially in relation to interface engineering, revealing insights into the dominant recombination pathways.^[^ ^38^ ^]^

(4) $$Aln\;\left(J_{0}\right)=\;Aln\left(J_{00}\right)-\frac{E_{a}}{kT}$$

In our analysis, *J*_00_ represents a pre‐factor dependent on recombination paths, while *E*_a_ signifies the activation energy for recombination. This energy can be deduced by plotting *A*ln(*J*_0_) against 1/*k*. Generally, by comparing the *E*_a_ value with the bandgap energy (*E*_g_) of the device, the main recombination path can be determined. *E*_a_ close to *E*_g_ demonstrates that bulk recombination is dominant; while *E*_a_ smaller than *E*_g_ means interface recombination is dominant. Therefore, the difference between *E*_a_ and *E*_g_ could determine the interface recombination degree. As shown in Figure 5c, the calculated *E*_g_ values for devices without and with MoO_3_ are 1.07 and 1.13 eV, respectively. Notably, the *E*_a_ of 1.13 eV for the device with MoO_3_ is closer to its *E*_g_ value (1.225 eV, Figure S4, Supporting Information), indicating that the bulk recombination is stronger than the interface recombination. This result demonstrated that the interface recombination was significantly passivated in flexible device with MoO_3_ interlayer. Electrochemical impedance spectroscopy (EIS) was conducted to examine charge recombination and transfer properties in the flexible Sb_2_Se_3_ devices. The recombination resistances (*R*_rec_) for both device types were derived from the arc's diameter in EIS Nyquist plots (Figure 5d), resulting in values of 27 846 Ω for devices without MoO_3_ and 43 847 Ω for those with MoO_3_. The higher *R*_rec_ in the latter suggests reduced carrier accumulation and recombination at the Sb_2_Se_3_/CdS and back contact interfaces, leading to improved open‐circuit voltage (*V*_OC_), fill factor (FF), and power conversion efficiency (PCE).

**Figure 6:**
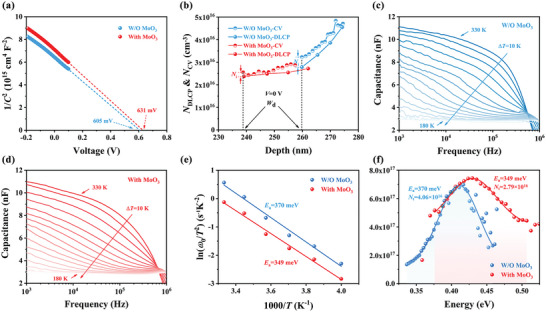
a) 1/*C* ^2^‐*V* plots, and b) *C*–*V* and DLCP profiles of the W/O MoO_3_ and With MoO_3_ flexible Mo‐foil Sb_2_Se_3_ devices. Admittance spectra of c) W/O MoO_3_ and d) With MoO_3_ Mo‐foil Sb_2_Se_3_ devices. e) Defect activation energies, and f) defect density spectra of the corresponding devices.

To further discern the decrease in interface defects in flexible Sb_2_Se_3_ devices, capacitance–voltage (*C*–*V*) and deep‐level capacitance profiling (DLCP) experiments were conducted. These investigations, detailed in Note S3 (Supporting Information), indicated an increase in *V*_OC_ for the device with MoO_3_, attributable to a raised built‐in voltage (*V*_bi_) from 0.605 to 0.631 V. This enhancement in *V*_bi_, as observed in the 1/*C* ^2^‐*V* plots (**Figures** **6**a and S6, Supporting Information), facilitates faster carrier transfer at the P–N junction and a reduction in trap states.^[^ ^39^ ^]^ To characterize interface defects, capacitance–voltage (*C*–*V*) and drive‐level capacitance analysis (DLCP) measurements were performed on W/O MoO_3_ samples and With MoO_3_ samples. It is known that the carrier doping density obtained from *C*–*V* profiling mainly represented responses from free carriers, bulk defects, and interfacial defects, whereas that obtained from DLCP profiling reflected responses only from free carriers and bulk defects.^[^ ^39^ ^]^ Therefore, the disparity between *C*–*V* and DLCP responses at zero bias is typically indicative of interface defect responses (*N*_i_), with estimated *N*_i_ values being significantly lower in devices with MoO_3_, as shown in Figure 6b. The various heterojunctions or interface‐associated parameters are summarized in **Table** **2**. This reduction suggests enhanced interface quality due to MoO_3_ treatment, leading to fewer carriers being trapped, thus benefiting carrier collection and improving FF.

**Table 2:** Summary of heterojunction and interface associated photovoltaic parameters of the Sb_2_Se_3_ devices.

Devices	*A*	*V*_TFL_ [V]	*N*_trap_ [cm^−3^]	*Φ*_B_ [meV]	*E*_a_ [eV]	*V*_bi_ [V]	*N*_i_ [cm^−3^]
Mo‐foil W/O MoO_3_	1.75	0.21	1.55 × 10^14^	449	1.07	0.605	4.52 × 10^15^
Mo‐foil With MoO_3_	1.54	0.19	1.40 × 10^14^	152	1.13	0.631	1.18 × 10^15^

Admittance spectroscopy (AS) measurements were also performed to probe the impact of MoO_3_ treatment on carrier trapping defects. The capacitance–frequency (*C*–*f*) curves for both device types were recorded across a temperature range of 180–330 K in 10 K increments (Figure 6c,d). Typically, high‐frequency capacitance is indicative of free carrier response, while low‐frequency capacitance suggests a response from deep traps and free carriers.^[^ ^40^ ^]^ In low temperature and high frequency scenarios, where the capacitance response converges, it's primarily governed by carrier freeze‐out affecting the devices geometric capacitance. Compared to the device without MoO_3_, the MoO_3_‐enhanced device showed a more stable capacitance response across frequencies, implying successful defect passivation by the MoO_3_ interlayer, as a less significant frequency dependency of capacitance correlates with lower defect densities in the absorber. The Arrhenius plots derived from the temperature‐dependent inflection frequency (*ω*_0_ = 2π*f*_max_) for both types of devices are presented in Figure 6e. The determination of the activation energy (*E*_a_) for defects was based on fitting this plot, as per the equation outlined in reference.^[^ ^41^ ^]^

(5) $$\omega_{0}=2\pi \nu_{0}T^{2}\exp\left(\frac{-E_{a}}{kT}\right)$$

In this section, “*k*” denotes the Boltzmann constant, “ν_0_” is defined as the attempt‐to‐escape frequency, “*ω*_0_” represents the inflection point frequency, and “*E*_a_” is the activation energy of the defect. Notably, the MoO_3_ interlayer's introduction does not lead to additional defects, as evidenced by the similar temperature‐dependent behavior and activation energies in both sets of devices at lower temperatures. The calculated activation energy values are 370 and 349 meV for the devices without and with MoO_3_, respectively. This small difference in activation energy (Δ*E*_a_ = 21 meV) suggests the presence of similar defect types in both cell types. Based on prior research and theoretical calculations,^[^ ^33^, ^40^ ^]^ we hypothesize that the dominant intrinsic defects in these Sb_2_Se_3_ devices include Se interstitials (Se_i_), Se_Sb_ antisites, and Sb vacancies (*V*_Sb_). However, pinpointing which of these defects is detected in admittance spectroscopy measurements remains challenging. Therefore, we preliminarily consider these three as the primary defect types in our flexible devices. The defect densities were analyzed using the Kimerling model and fitted to a Gaussian distribution.^[^ ^42^ ^]^

(6) $$E\;\left(\omega \right)=kT\ln\left(\frac{2\pi \upsilon_{0}T^{2}}{\omega }\right)$$

(7) $$N_{t}\;\left(E\left(\omega \right)\right)=-\frac{V_{d}}{q\omega }\frac{dC}{d\omega }\frac{\omega }{kT}$$

In this section, *N*_t_(*E*(*ω*)) denotes the defect density, *V*_d_ is the P–N junction's built‐in voltage, *E* represents the energy distance between the defect level and the valence or conduction band maximum, and *ω* is the angular frequency. The defect densities for devices without and with the MoO_3_ interlayer are 4.06 × 10^16^ cm^−3^ eV^−1^ and 2.79 × 10^16^ cm^−3^ eV^−1^, respectively, as shown in Figure 6f. The reduced activation energy *E*_a_ value post‐MoO_3_ introduction indicates a faster hole emission rate, likely minimizing recombination center activity. The decrease in defect density is beneficial, potentially reducing the open‐circuit voltage (*V*_OC_) deficit. These results, combined with capacitance‐voltage (*C*–*V*), deep‐level capacitance profiling (DLCP), and admittance spectroscopy (AS) analyses, suggest that MoO_3_ enhances not just the back contact but also the overall quality of the heterojunction and absorber layer, improving the flexible Sb_2_Se_3_ device's performance.

**Figure 7:**
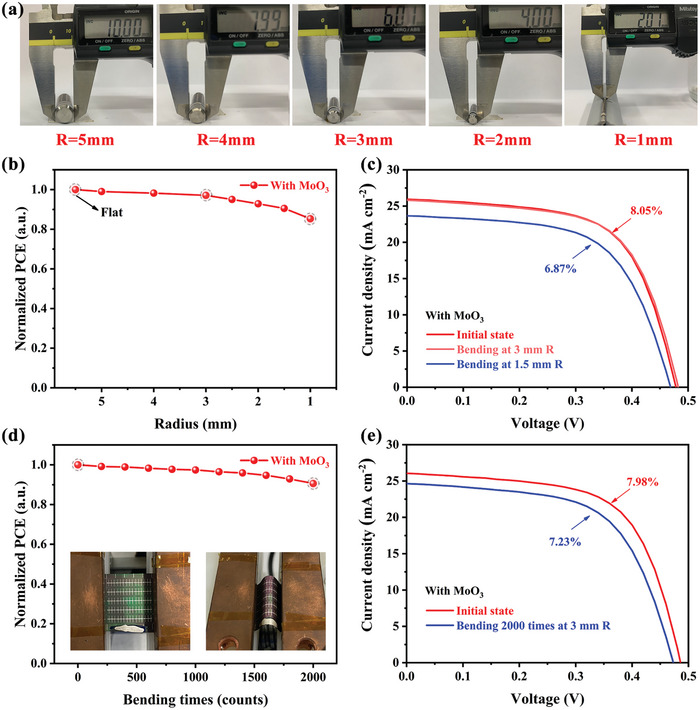
a) Photographs of flexible Sb_2_Se_3_ solar cells at varied bending radii. b) Normalized PCE degradation at different bending *R* (Flat to 1 mm). c) *J*–*V* curves of the flexible Sb_2_Se_3_ solar cells at initial state and bending at a 3 and 1 mm *R*. d) Normalized PCE evolution with different bending cycles (0‐2000 times) at a fixed 3 mm *R*. Inset: the schematic diagram of the bending equipment and the bending state of the flexible Sb_2_Se_3_ solar cells. e) *J*–*V* curves of the flexible Sb_2_Se_3_ solar cells at initial state and after bending at 3 mm *R* for 2000 times.

The flexibility and deformability of these devices, crucial for various applications, are attributable to Sb_2_Se_3_’s unique 1D crystal structure. Post‐bending, the strain in different layers and the entire device can be calculated using the neutral axis concept (Note S4 and Figure S7, Supporting Information). These flexible properties were tested on cylinders with varying radii, demonstrating remarkable flexibility and the ability to withstand significant bending while maintaining over 90% of initial performance even after 2000 bending cycles (**Figure** **7**). The detailed performance parameters of the devices before and after bending treatments are provided in Tables S2 and S3 (Supporting Information). This resilience is vital for practical applications, although performance does decline slightly under extreme bending, likely due to structural stresses.

## 3 Conclusion

In this study, we introduce a straightforward method to boost flexible Sb_2_Se_3_ solar cell performance via buried interface engineering. Incorporating a MoO_3_ interlayer between Mo and Sb_2_Se_3_ through magnetron sputtering significantly enhances the crystalline quality of Sb_2_Se_3_ films, encourages favorable growth orientation, lowers back contact barrier, and mitigates harmful defects. This results in notable improvements in open‐circuit voltage (0.479 V) and fill factor (64.93%), achieving a high efficiency of 8.23%. Moreover, these cells display exceptional flexibility and durability, retaining over 90% efficiency even after 2000 bending cycles at a 3 mm radius. This approach offers a promising avenue for enhancing flexible Sb_2_Se_3_ photovoltaic devices.

## 4 Experimental Section

### Device Fabrication

Molybdenum (Mo) foil substrates, 0.03 mm in thickness, were initially cleansed using a detergent, ethanol, and deionized water sequence in an ultrasonic bath for 10 min. A back contact layer of Mo, ≈1 µm thick, was then sputtered onto these foils using direct current (DC) magnetron sputtering. To improve device adhesion on the flexible substrate and the compactness of the Mo film, sputtering was conducted at room temperature under alternating low (1.5 Pa) and high (0.3 Pa) pressures. Next, An MoO_3_ interlayer, varying in thickness between 5, 15, or 30 nm, was subsequently applied on the bi‐layer of Mo using RF magnetron sputtering at a base pressure below 8 × 10^−4^ Pa. Specifically, during the sputtering process of the MoO_3_ layer, only pure Ar was utilized without any injection of O_2_, the Ar gas flow rate was 30 sccm, the working pressure was maintained at 0.4 Pa with the substrate at room temperature, the sputtering power density was 1.59 W cm^−2^, the deposition rate was ≈4 nm min^−1^, and the film thickness was regulated by adjusting the sputtering duration. Following this, Sb precursor layers were deposited onto the MoO_3_ film via magnetron sputtering, based on an optimized process detailed in previous research.^[^ ^43^ ^]^ Post‐deposition, a heat treatment for post‐selenization was applied to facilitate an in‐situ combination reaction and self‐assembled growth of high‐crystallinity Sb_2_Se_3_ thin films. The Sb_2_Se_3_ surface was then coated with a CdS buffer layer using the Chemical Bath Deposition (CBD) method, achieving a thickness of ≈70 nm. The indium tin oxide (ITO) window layer was sputtered using RF sputtering at 100 W, 0.35 Pa pressure, with argon gas flow at 30 sccm and oxygen gas at 5 sccm. The final step involved the thermal evaporation of an Ag grid electrode onto the ITO layer, using a deposition mask to define an isolated region measuring 0.16 cm^2^ (active area was 0.14 cm^2^). Consequently, the complete flexible Sb_2_Se_3_ devices were structured as Mo‐foil/Mo/MoO_3_/Sb_2_Se_3_/CdS/ITO/Ag. Figure 1 illustrates the schematic diagram of the Sb_2_Se_3_ solar cell fabrication process.

### Characterizations

The surface and cross‐sectional morphology of Sb_2_Se_3_ thin films and devices were examined using a Scanning Electron Microscope (SEM, Zeiss SUPRA 55). X‐ray Diffraction (XRD, Ultima‐iv) analysis, conducted at 40 kV and 40 mA using Cu Kα radiation, assessed the crystallization properties of Sb_2_Se_3_. The current density‐voltage (*J*–*V*) characteristics of flexible Sb_2_Se_3_ devices were measured under AM 1.5G simulated sunlight (100 mW cm^−2^, 25 °C). External Quantum Efficiency (EQE) spectra were evaluated using the Zolix SCS101 system, coupled with a Keithley 2400 source meter. Dark *J*–*V* temperature dependence (*J*–*V*–*T*) was analyzed using a Lakeshore 325 temperature controller system. Electrochemical Impedance Spectroscopy (EIS) of the flexible Sb_2_Se_3_ device was performed on a CHI600E electrochemical workstation, with frequency range settings from 1 Hz to 1 MHz. Capacitance‐voltage (*C*–*V*) measurements were executed at room temperature in darkness, using a 10 kHz frequency and a sweeping DC bias voltage from −0.5 to 0.1 V, alongside a 30 mV AC amplitude. Drive‐Level Capacitance Profiling (DLCP) involved a DC bias range of −0.2–0.2 V and an AC modulation amplitude of 0.02–0.14 V, within a frequency range of 100 HZ–1 MHz at temperatures between 180–330 K (Lake Shore, 325 cryogenic temperature controller). Admittance Spectroscopy (AS) measurements (Victor, Digital LCR meter) were also conducted over a frequency range of 100–1 MHz, at temperatures from 180 to 330 K (Lake Shore, 325 cryogenic temperature controller).

## Conflict of Interest

The authors declare no conflict of interest.

## Supporting information

Supporting Information

Supporting Information

## Data Availability

The data that support the findings of this study are available from the corresponding author upon reasonable request.
