# Supplementary material for: Back Interface and Absorber Bulk Deep‐Level Trap Optimization Enables Highly Efficient Flexible Antimony Triselenide Solar Cell
Source: Adv Sci (Weinh). 2024 Mar 20;11(22):2310193. doi: 10.1002/advs.202310193 (PMC11165550; doi:10.1002/advs.202310193)
Supplement: Supplementary file 1 — Supporting Information [file ADVS-11-2310193-s001.pdf]

ADVANCED SCIENCE
Open Access

Supporting Information

for *Adv. Sci.*, DOI 10.1002/advs.202310193

Back Interface and Absorber Bulk Deep-Level Trap Optimization Enables Highly Efficient Flexible Antimony Triselenide Solar Cell

Jia Yang, Mingdong Chen*, Guojie Chen, Yanqing Hou, Zhenghua Su, Shuo Chen, Jun Zhao and Guangxing Liang**

Supporting Information

Back Interface and Absorber Bulk Deep-Level Trap Optimization Enables Highly Efficient Flexible Antimony Triselenide Solar Cell

Jia Yang, Mingdong Chen*, Guojie Chen, Yanqing Hou, Zhenghua Su, Shuo Chen, Jun Zhao, Guangxing Liang**

Supplementary Note1 Texture coefficient calculation

The texture coefficients (*TC*) were calculated based on the following equation to evaluate the dominant [hk1] growth orientation more intuitively:[1]

$$TC_{\mathrm{hkl}} = \frac{I_{(\mathrm{hkl})}}{I_{0(\mathrm{hkl})}} / \left(\frac{1}{N}\sum_{i=1}^{N}\frac{I_{(\mathrm{h}_i\mathrm{k}_i\mathrm{l}_i)}}{I_{0(\mathrm{h}_i\mathrm{k}_i\mathrm{l}_i)}}\right), \quad \text{(S1)}$$

where $I_{(\mathrm{hkl})}$ and $I_{0(\mathrm{hkl})}$ are the diffraction peak intensities of [hkl] planes in the measured and standard XRD pattern (JCPDS 15-0861) of Sb_2Se_3, respectively. The N is the total number of the diffraction peaks in the measured XRD pattern. A large *TC* value of a diffraction peak indicates a preferred orientation along this direction.

Supplementary Note2 Reverse saturation current density (J_0) and the diode ideality factor (A) calculations

The *J-V* behavior of the p-n junction can be expressed by the following equation:[2,3]

$$J = J_0 \exp\left[\left(\frac{q}{AkT}\right)(V - JR_{\mathrm{s}})\right] + GV - J_{\mathrm{L}} \quad \text{(S2)}$$

where G, R_s, A, and J_0 represent the shunt conductance, series resistance, ideality factor, and

reverse saturation current, respectively, k represents the Boltzmann constant, q represents the elementary charge on the electron and J_L is the light current density. The values of G were estimated from the flat portions under reverse bias of plot of dJ/dV against V. The plots of dV/dJ in relation to $(J+J_{SC})^{-1}$ are presented in **Figure 4**b. The intercept of the y-axis provides the series resistance R, while the slope corresponds to AkT/q, allowing for determination of the diode ideality factor A. Finally, the reverse saturation current J_0 were extracted from the plot of $\ln(J+J_{SC}-GV)$ against $V-RJ$.

Supplementary Note3 *C-V* and DLCP analysis

To evaluate the defect density of the Sb_2Se_3 absorber layers more clearly and the combination properties of the solar cells, *C-V* and DLCP experiments were conducted. The following equations can be employed to represent the $N_{C\text{-}V}$ and N_{DLCP} graphs against the profiling depth:[4]

$$N_{C-V} = \frac{-2\varepsilon_{r,n}N_D}{\left(\frac{d\left(\left(1/C^2\right)\right)}{dV}\right)qA^2\varepsilon_0\varepsilon_{r,n}\varepsilon_{r,p}N_D+2\varepsilon_{r,p}} \quad \text{(S3)}$$

$$N_{DLCP} = -\frac{C_0^3}{2q\varepsilon_0\varepsilon_{r,p}A^2C_1} \quad \text{(S4)}$$

$$x = \frac{\varepsilon_0\varepsilon_{r,p}A}{C_0} \quad \text{(S5)}$$

where N_D is the doping density of CdS, A is the device area, ε_0, $\varepsilon_{r,n}$ and $\varepsilon_{r,p}$ represents the permittivity of free space, the relative permittivity of CdS and Sb_2Se_3, respectively. C_0 and C_1 are two quadratic fitting parameters determined from the C-V curves. In theory, the DLCP-measured doping density (N_{DLCP}) only discloses the responses from free carriers and bulk defects, while the *C-V* determined doping density ($N_{C\text{-}V}$) represents the responses from free

carriers, bulk defects, and interfacial defects.[4] As a result, the difference between *C*-*V* and DLCP profiling at zero bias can be utilized for calculating the interface defect density (N_i).

Supplementary Note4 The neutral axis analysis of the flexible devices

The neutral axis (NA) is the key factor to estimate the strain of device under bending states. The locations of NA (Z_{NA}) is determined by the total force in the cross section, where the strain in the film is zero. The relationship between Z_{NA} and strain under different curvature radii (R) is shown in the following equation.[5,6]

$$z_{NA} = \frac{\sum_{k=1}^{n} E_k^* t_k^* z_k}{\sum_{k=1}^{n} E_k^* t_k^*} \tag{S6}$$

$$E_k^* = \frac{E_k}{1-\nu_k^2} \tag{S7}$$

$$\varepsilon(z,R) = \frac{z-z_{NA}}{R} \tag{S8}$$

Where E_k, ν_k and t_k are Young's modulus, Poisson's ratio and thickness of each layer, respectively, and z_k is the coordinate of half thickness film. Then we can obtain the R dependent strain (ε_z). All calculation parameters are listed in **Table S**1.

Supplementary Figures

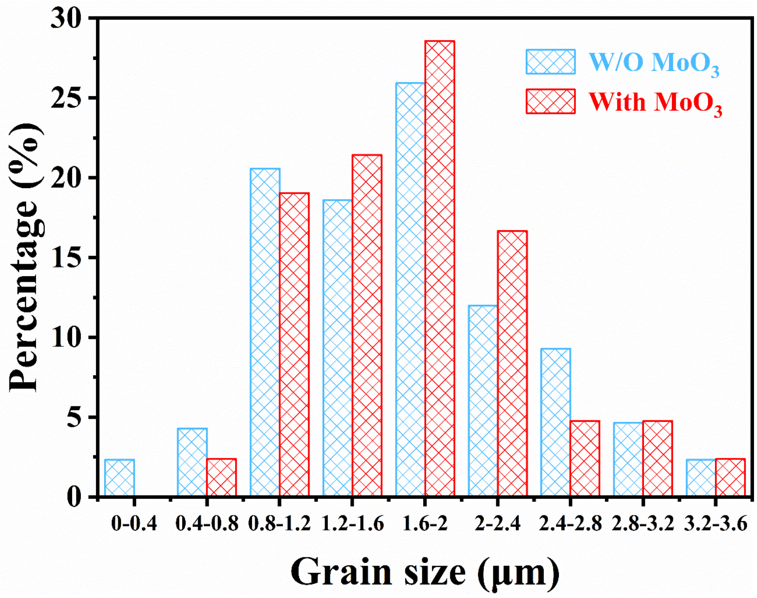

Figure S1. Crystal size distribution diagram of Sb_2Se_3 thin films without MoO_3 layer and with MoO_3 layer.

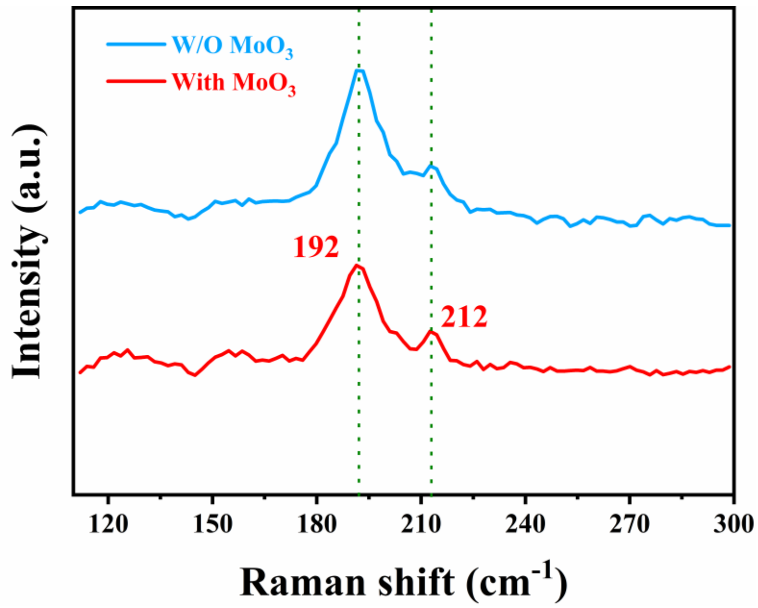

Figure S2. Raman spectra of Sb_2Se_3 thin films without MoO_3 interlayer and with MoO_3 interlayer.

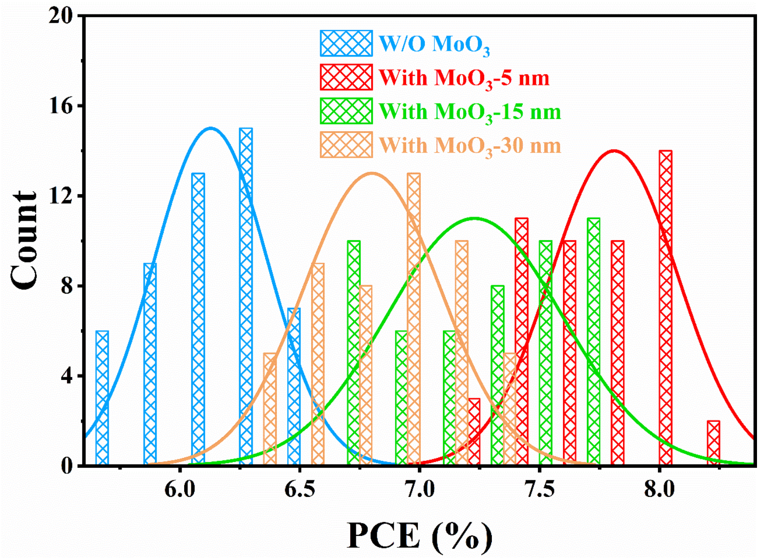

Figure S3. The statistical PCE distribution histograms of the flexible Sb_2Se_3 devices with different MoO_3 interlayer thicknesses.

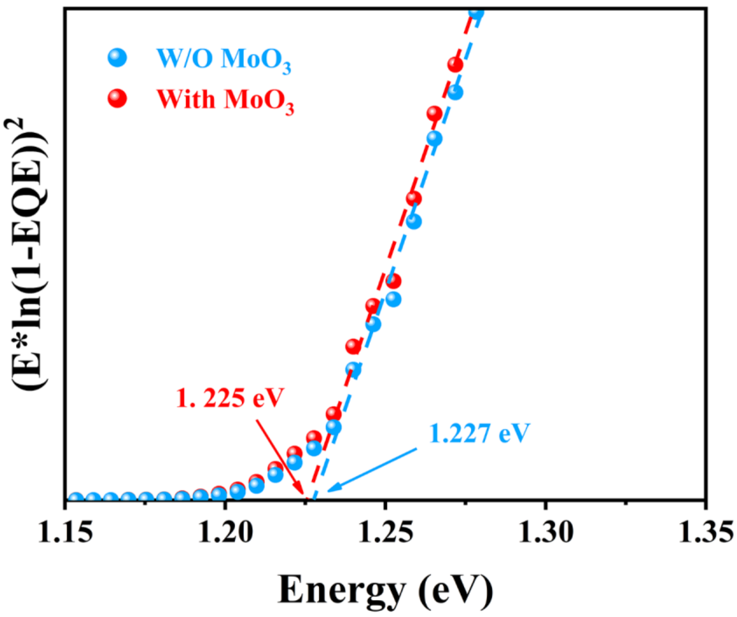

Figure S4. The bandgap (E_g) of W/O MoO_3 and With MoO_3 devices extracted from EQE data.

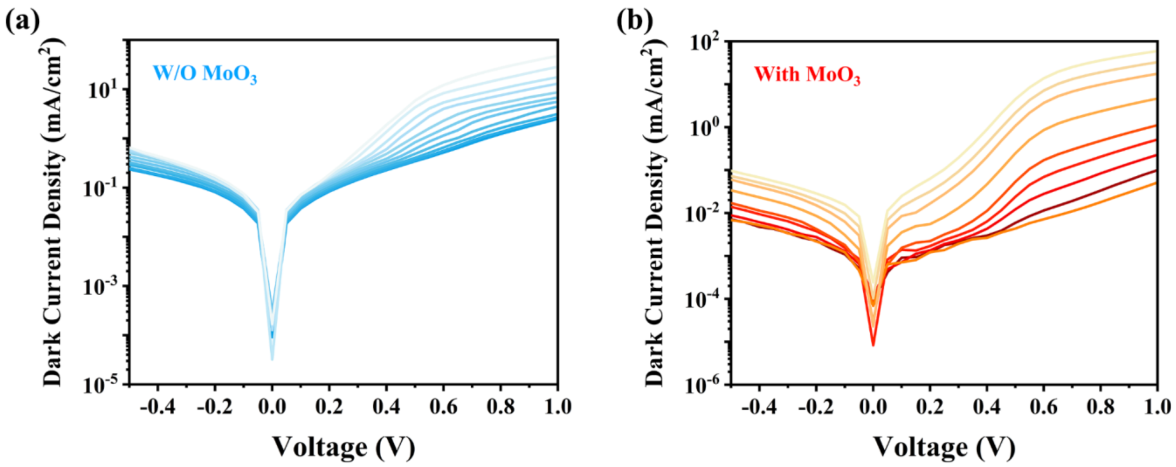

Figure S5. *J*-*V*-*T* plots of (a) W/O MoO_3 device and (b) With MoO_3 device.

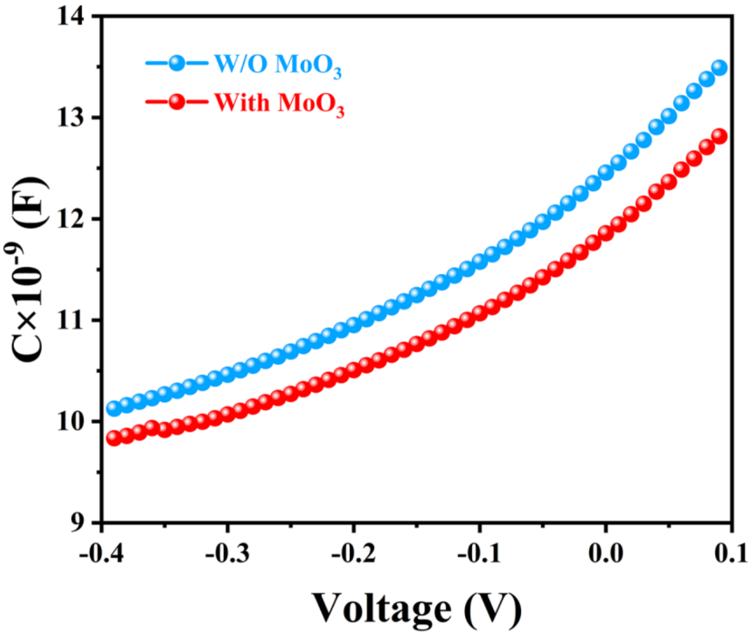

Figure S6. *C*-*V* curves of W/O MoO_3 and With MoO_3 flexible Sb_2Se_3 devices.

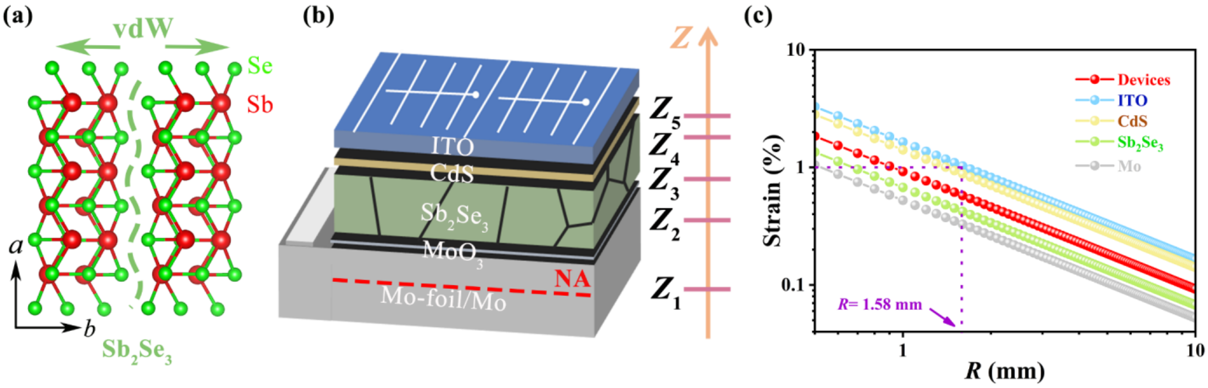

Figure S7. (a) The crystal structure of Sb_2Se_3. The light green dotted line mark the gap between $(Sb_4Se_6)_n$ ribbons. (b) Schematic diagram of the neutral axis (NA) in flexible Sb_2Se_3 device. Z_k stands for the center position of each layer. NA of the whole device is marked by a red dash line. (c) The strain of the whole device, ITO, CdS, Sb_2Se_3 and Mo layers as a function of radius.

Supplementary Tables

Table S1. The thickness, Young modulus and Poisson ratio of each layers.

Layer	Thickness (μm)	Young modulus (Gpa)	Poisson ratio
Mo-foil	30	329	0.31
MoO_3	0.005	-	-
Mo	1	329	0.31
Sb_2Se_3	1.4	83	0.26
CdS	0.07	68	0.34
ITO	0.4	135	0.27

Table S2. The performance parameters of the flexible Sb_2Se_3 solar cell bending at different *R*.

Bending radius (mm)	V_{OC} (V)	J_{SC} (mA/cm^2)	FF (%)	PCE (%)
Flat	0.478	25.95	64.92	8.05
5	0.480	25.89	64.13	7.97
4	0.480	25.85	63.72	7.91
3	0.481	25.82	62.71	7.79
2	0.476	25.04	62.68	7.47
1.5	0.471	24.73	62.49	7.28
1	0.469	23.66	61.92	6.87

Table S3. The performance parameters of the flexible Sb_2Se_3 solar cells after different bending cycles.

Bending circles	V_{OC} (V)	J_{SC} (mA/cm^2)	FF (%)	PCE (%)
0	0.485	26.07	63.11	7.98
400	0.484	25.75	62.83	7.83
800	0.483	25.70	62.75	7.79
1200	0.479	25.65	62.67	7.70

1600	0.475	25.50	62.41	7.56
2000	0.474	24.65	61.87	7.23

References

[1] G. Liang, M. Chen, M. Ishaq, X. Li, R. Tang, Z. Zheng, Z. Su, P. Fan, X. Zhang, S. Chen, *Adv. Sci.* **2022**, *9*, 2105142.

[2] X. Wen, Z. Lu, G.-C. Wang, M.A. Washington, *Nano Energy* **2021**, *85*, 106019.

[3] G.-X. Liang, Y.-D. Luo, S. Chen, R. Tang, Z.-H. Zheng, X.-J. Li, X.-S. Liu, Y.-K. Liu, Y.-F. Li, X.-Y. Chen, Z.-H. Su, X.-H. Zhang, H.-L. Ma, P. Fan, *Nano Energy* **2020**, *73*, 104806.

[4] Y. Luo, G. Chen, S. Chen, N. Ahmad, M. Azam, Z. Zheng, Z. Su, M. Cathelinaud, H. Ma, Z. Chen, P. Fan, X. Zhang, G. Liang, *Adv. Funct. Mater.* **2023**, *33*, 2213941.

[5] C. Chen, K. Li, F. Li, B. Wu, P. Jiang, H. Wu, S. Lu, G. Tu, Z. Liu, J. Tang, *ACS Photonics* **2022**, *7*, 352.

[6] K. Li, F. Li, C. Chen, P. Jiang, S. Lu, S. Wang, Y. Lu, G. Tu, J. Guo, L. Shui, Z. Liu, B. Song, J. Tang, *Nano Energy* **2021**, *86*, 106101.
